# Influence of the human papillomavirus on the radio-responsiveness of cancer stem cells in head and neck cancers

**DOI:** 10.1038/s41598-020-59654-4

**Published:** 2020-02-17

**Authors:** Paul Reid, Alexander H. Staudacher, Loredana G. Marcu, Ian Olver, Leyla Moghaddasi, Michael P. Brown, Eva Bezak

**Affiliations:** 1 School of Health Sciences, University of South Australia, Adelaide, SA 5001 Australia; 2 Cancer Research Institute, University of South Australia, Adelaide, SA 5001 Australia; 3 Faculty of Science, University of Oradea, Oradea, 410087 Romania; 4 Department of Physics, University of Adelaide, Adelaide, SA 5005 Australia; 5 Department of Medical Physics, GenesisCare, Adelaide, SA 5000 Australia; 6 Translational Oncology Laboratory, Centre for Cancer Biology, SA Pathology and University of South Australia, Adelaide, SA 5000 Australia; 7 School of Medicine, University of Adelaide, Adelaide, SA 5000 Australia; 8 Cancer Clinical Trials Unit, Royal Adelaide Hospital, Adelaide, SA 5000 Australia

**Keywords:** Biophysics, Cancer

## Abstract

A growing proportion of head and neck cancers (HNC) result from HPV infection. Between HNC aetiological groups (HPV positive and HPV negative) clinical evidence demonstrates significantly better treatment response among HPV positive cancers. Cancer stem cells (CSCs) are identified in HNC tumour populations as agents of treatment resistance and a target for tumour control. This study examines dynamic responses in populations of a CSC phenotype in HNC cell lines following X-irradiation at therapeutic levels, and comparing between HPV statuses. Variations in CSC density between HPV groups showed no correlation with better clinical outcomes seen in the HPV positive status. CSC populations in HPV positive cell lines ranged from 1.9 to 4.8%, and 2.6 to 9.9% for HPV negative. Following 4 Gy X- irradiation however, HPV negative cell lines demonstrated more frequent and significantly greater escalation in CSC proportions, being 3-fold that of the HPV positive group at 72 hours post irradiation. CSC proportions of tumour populations are not fixed but subject to change in response to radiation at therapeutic dose levels. These findings imply a potential effect of aetiology on radio-responsiveness in CSCs, illustrating that clonogen treatment response may be more informative of therapy outcomes than inherent population density alone.

## Introduction

Head and neck cancers are invasive carcinomas of the mucosal epithelium involving the complex anatomy of this area and frequently requiring radiotherapy as part of an approach aimed at tumour control whilst conserving normal tissue function. Globally, around 3.8% of cancers are head and neck cancers of which approximately 95% are squamous cell carcinomas (HNSCC)^1,2^. Two principal aetiologies of HNSCC are those cancers associated with agents such as tobacco and alcohol and those resulting from the human papillomavirus (HPV) infection, which displays an anatomical preference for the base of tongue and tonsils, resulting in oropharyngeal squamous cell carcinoma (OPSCC)^3^. The most important distinction between these aetiologies is the significantly better treatment responses shown by HPV positive HNSCC, where the 5-year overall survival rate is around 80% compared to 50% for HPV negative HNSCC^4,5^. Moves toward more individualised treatment for HNSCC patients in terms of HPV status are hampered however, largely because the mechanisms and predictability of different treatment sensitivities according to HPV status remaining poorly understood^6–8^.

Cancer stem cells (CSCs) are a small but critical subset within tumour cell populations, having unlimited capacity for self-renewal and generation of differentiated progeny. CSCs are mediators of metastases and recurrence following therapy and the most treatment resistant of tumour cells^9^. They demonstrate changes in divisional dynamics that can accelerate tumour re-population and are the numerical beneficiaries of tumour plasticity whereby de-differentiation of non-CSCs further increases the CSC population^10–14^. Consequently, therapy needs to account for CSC elimination to achieve tumour control^15,16^. CSCs have been identified in HNSCC tumour populations by several cellular markers tested by serial xenotransplantation of which CD44 and aldehyde dehydrogenase (ALDH) are found to be markers of stemness in both HPV statues^17–21^. Density of tumour CSC populations is recognised as a strong prognostic marker in a range of cancers^22^ but evidence of CSC density in HNSCC, by HPV status, is conflicting and fails to correlate with the better treatment outcomes of the HPV positive group^23–25^. These mixed results strongly imply additional factors are involved in CSC treatment response among HNSCC that result from HPV infection, and the prognostic interpretation of tumour CSC density may depend on HNSCC aetiology. Greater radiosensitivity and limited DNA repair following irradiation has been demonstrated in HPV positive tumours in pre-clinical studies^7,26–28^. Differences in the response to treatment according to HPV status in HNSCC has been widely researched, but data on the role played by CSCs, in terms of their HPV status, is scarce. What role CSCs play, as a result of HPV status, in response to treatment through radioresistance or the plasticity of tumour populations to replace them, remains unclear.

The aim of this study has been to investigate behavioural responses of CSCs to irradiation, in terms of their HPV status, measuring inherent CSC proportions of HNSCC cell lines and changes in CSC populations in response to irradiation as a function of HPV status. The key focus of this *in vitro* study was on the quantitative evaluation of cell line dynamics during fractionated radiotherapy among HPV positive and negative aetiologies alike. Our previous work in this area, as a pilot study, examined only one cell line each HPV status, as such being insufficient to draw comparison between aetiologies, but suggestive of need for further investigation^29^. The current work comprises of data from 6 cell lines and provides valuable additional information, enabling not only qualitative but also quantitative comparison between HNSCC aetiologies by an examination of dynamic responses among CSCs to therapeutic levels of radiation in terms of their HPV status. While general understanding of HPV positive and negative responses are known, dynamic quantitative data for up to 6 cell lines has not been reported in literature so far^30^. This study therefore progresses current knowledge from generic toward specific where findings demonstrate significant differences in CSC population response to X-ray irradiation between the HPV groups, implying greater repopulating ability in HPV negative HNSCC.

## Materials and Methods

This *in vitro* experimental study measured a) the inherent proportions of the CSC phenotype CD44+/ALDH + in HNSCC cell lines, b) changes in CSC proportions of population over time following 4 Gy X-ray dose, c) parallel assays to compare cell population response in T25 and 6 well plates (Cellstar: Greiner bio-one, Frickenhausen, Germany).

### Cell culture

Six HNSCC cell lines were selected, 3 of each HPV status. UM-SCC-1, UM-SCC-17a, UM-SCC-22a and UM-SCC-47 were purchased through Merck Millipore (Darmstadt, Germany). UPCI-SCC-090 (ATCC CRL-3239TM) and UPCI-SCC-154 (ATCC CRL-3241TM), both HPV positive, were purchased from ATCC (Manassas VA). UM-SCC-1, UM-SCC-17a and UM-SCC-22a are HPV negative and UM-SCC-47, UPCI-SCC-090 and UPCI-SCC-154 are HPV positive (Table 1). Authenticity of cell lines at purchase was established by STR profile by the suppliers and cell lines analysed within 12 passages. Status of TP53 mutation among these cell lines reflects the common characteristics of HPV status whereby the 3 HPV negative cell lines have TP53 mutations and the HPV positive lines are wild type^31^. Cells were cultured in T25 flasks using Dulbecco’s Modified Eagle’s Medium with 4500 mg/L glucose, (Sigma-Aldrich Darmstadt, Germany) supplemented with with 10 mM HEPES, 10% foetal bovine serum (FBS), 100 U/mL penicillin and with 0.1 mg/mL streptomycin (Sigma-Aldrich Darmstadt, Germany). Cells were incubated in humidified atmosphere of 5% CO_2_ at 37 °C. Cell lines were tested for mycoplasma used MycoAlert detection kit (Lonza Group Ltd Basel, Switzerland) and were negative.

**Table 1:** HNSCC cell line characteristics^39–41^.

Cell line	HPV status	Anatomy	Gender	Age	TP53 mutation
UM-SCC-47	Positive	Lateral tongue	M	53	Wild Type
UPCI-SCC-090	Positive	Base of tongue	M	46	Wild type
UPCI-SCC-154	Positive	Tongue	M	54	Wild type
UM-SCC-1	Negative	Floor of mouth	M	73	Splice site
UM-SCC-17a	Negative	Laryngeal	F	48	Missense
UM-SCC-22a	Negative	Hypopharynx	F		Missense

### Irradiation setup

Cell cultures were irradiated in T25 flasks using a 160 kV, 25 mA, RS2000 X-ray cabinet irradiator (Rad Source, Buford GA) calibrated using the AAPM TG61 protocol^32^ to deliver 4 Gy to the cell monolayer whilst flasks were encased in paraffin wax and mounted on 7 cm of solid water (RW3; PTW, Freiburg DE; ρ = 1.0459 g/cm^3^) to achieve full scatter conditions. Flasks were filled with media (21 mm depth over the cell monolayer) to achieve electronic equilibrium at the cell layer. One T25 flask of each cell line, for each analysis time point (24, 48 and 72 h) was irradiated with a 4 Gy dose. 4 Gy was chosen as the treatment dose as it is low enough to be at the upper end of what a single fractionated dose might be in therapy, and sufficiently high to produce cell killing and a clear response among survivors. Matching controls for each flask were sham irradiated. Media was changed post irradiation and flasks for assay were incubated with cells remaining *in situ* until analysis.

### Flow cytometry/Immunocytochemistry

**Figure 1:**
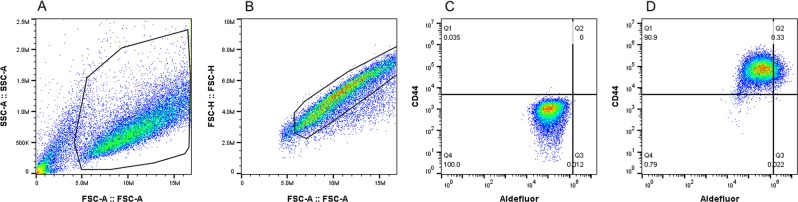
Gating strategy for cell counting by flow cytometry. (**A**) Initial selection of viable cells by forward vs side scatter. (**B**) Selection of single cells from gated area in A, by forward scatter-area vs height. (**C**) Gating by negative controls for ALDH and CD44. (**D**) CD44+/ALDH + cells in upper right quadrant. FloJo Software v10.0.7 Ashland, www.FlowJo.com.

CSCs were putatively identified in the 6 HNSCC cell lines by 2 cellular markers: cluster of differentiation 44 (CD44), a cell surface protein and adhesion molecule, and aldehyde dehydrogenase (ALDH), a metabolic enzyme. CD44 expression was determined by anti-mouse/human CD44 antibody, clone IM7, fluorophore APC (BioLegend, San Diego CA). ALDH expression was measured using Aldefluor (StemcellTM Technologies, Vancouver BC). Gating for CD44 was based on the APC isotype control and diethylaminobenzaldehyde (DEAB) was used for gating of ALDH expression (Fig. 1). Cells were stained as per manufacurer’s protocol and analysed using a BD Accuri C6 flow cytometer with at least 20,000 cells analysed (BD & Co. Franklin Lakes, NJ). 7-Aminoactinomycin D (7-AAD) (Sigma Aldrich, St Louis MO) was used to exculude non-viable cells.

Baseline proportions of CSC populations were established through concurrent flow cytometry of the 6 unirradiated cell lines for their CD44+/ALDH + population. Measurements of CSC proportions in unirradiated and irradiated cell line populations were conducted 7 times at 24, 48 and 72 hours following a 4 Gy X-ray dose. Time points at 1, 2 and 3 days were chosen as they reflect normal intervals between radiotherapy fractions during which CSC density may increase or potentially re-establish populations.

### Validation by protocol comparison

To test for associative effect on CSC proportions as a result of cells being contained *in situ* following irradiation until analysis, parallel assays were conducted to test for significance of any difference in results. For comparison, cells in T75 flasks, also under 21 mm of media and associated sham irradiated controls, were irradiated with 4 Gy then detached using TrypLE Select (Thermo Fisher, Waltham MA) centrifuged and re-plated in 6 well plates until concurrent analysis with cells from T25 flasks.

### Statistical analysis

Flow cytometry data was analysed in Microsoft Excel and Prism v7.01 (GraphPad Software Inc. La Jolla CA). Data from repeated cell line analysis (n = 7) was reported as mean and SEM for each cell line. Multiple t-tests were used to determine significance in CSC population change following 4 Gy dose. Two way ANOVA and Sidak’s multiple comparisons were used to test significance for difference in ratio of response. Unpaired t-tests were used to compare CSC data for control cells of T25 versus 6 well plate methods and irradiated cells. Significance considered to be p < 0.05 (* =  < 0.05, ** =  < 0.01, *** =  < 0.001, **** =  < 0.0001).

## Results

### Baselines CSC-like populations

**Figure 2:**
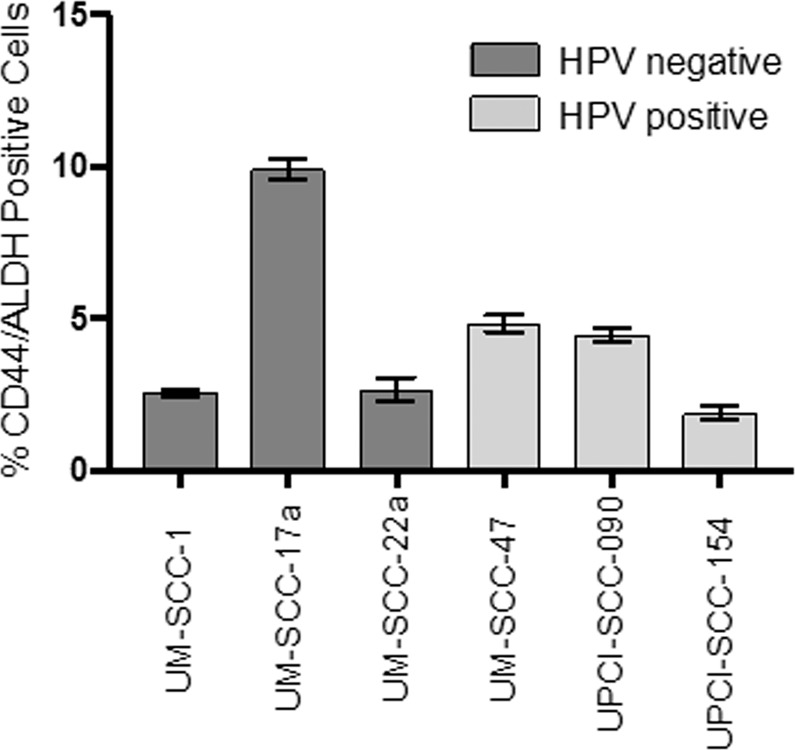
Baseline proportions of CSC population in HNSCC cell lines by HPV status. Analysis repeats n = 7. Error bars by standard error of mean. GraphPad Prism v8.2.1 www.graphpad.com.

Proportions of putative CSCs in untreated cultures of 6 HNSCC lines were determined by staining for CD44 and ALDH. The proportions of CSC varied between 2% to 10% of the total cell population. Large variations in CSC density was observed among the cell lines, irrespective of HPV status (Fig. 2).

### Cell line response to 4 Gy X-ray by CSC proportion

**Figure 3:**
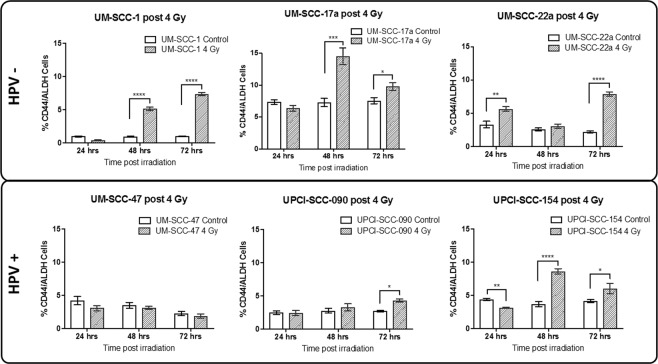
CSC proportions in treated cells and controls across 3 days following 4 Gy X-ray dose. Analysis repeats n = 7. Error bars by standard error of mean. GraphPad Prism v8.2.1 www.graphpad.com.

The HPV negative cell lines demonstrated greater frequency of significant elevations in CD44+/ALDH + population density following Gy irradiation. Additionally, higher levels of significance were observed in the HPV negative cell lines with the average increase being 2-fold that of the HPV positive group across 3 time points (Fig. 3). Of the 6 cell lines, only HPV positive UM-SCC-47 did not show any significant changes in CD44+/ALDH + proportions in the irradiated population across the 3 days following irradiation. HPV negative UM-SCC-1 showed the most significant responses with 5.3 and 7.1-fold increases at 48 and 72 hours respectively. Only UM-SCC-22a demonstrated an increase at 24 hours. For the other cell lines, elevation in the CD44+/ALDH + proportion came later at 48 and 72 hours post irradiation.

### Ratio of response by HPV status

**Figure 4:**
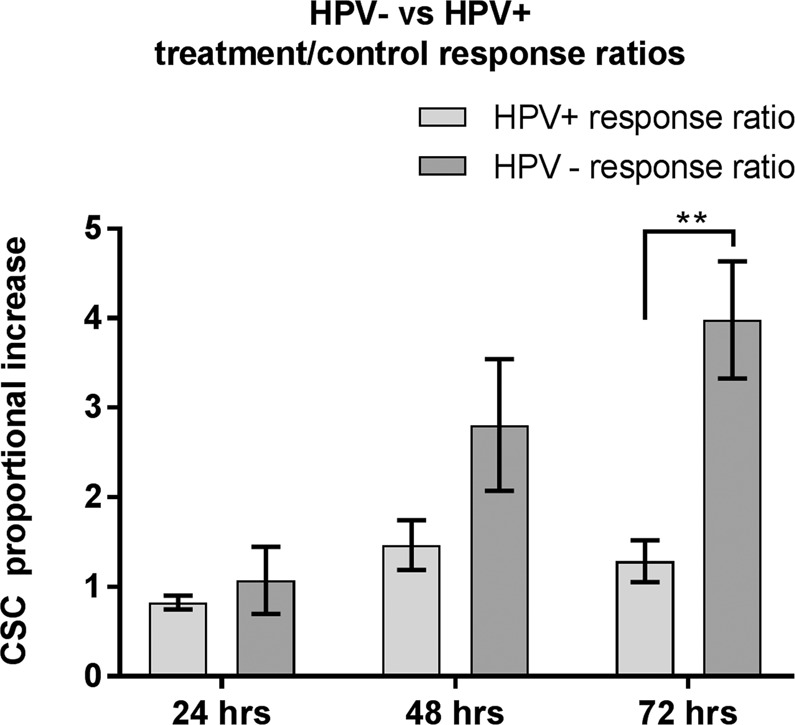
Ratio of response in CSC population following 4 Gy by HPV status. Analysis repeats n = 7. Error bars by standard error of mean. GraphPad Prism v8.2.1 www.graphpad.com.

When analysing the response to 4 Gy by ratio of CSC proportion in treated cells to controls, in terms of HPV status, the HPV negative cell lines as a group demonstrated an increasing escalation in the CSC proportion compared to the HPV positive cell lines. This difference showed the most significance (p = 0.0038) at 72 hours following irradiation (Fig. 4). The latency of escalation in CSC populations within these time points reflects the growing difference in proportional response according to HPV status whereby the increase in CD44+/ALDH + numbers in the HPV negative group is 3.1-fold that of the HPV positive group at 72 hours.

### Validation by protocol comparison

**Figure 5:**
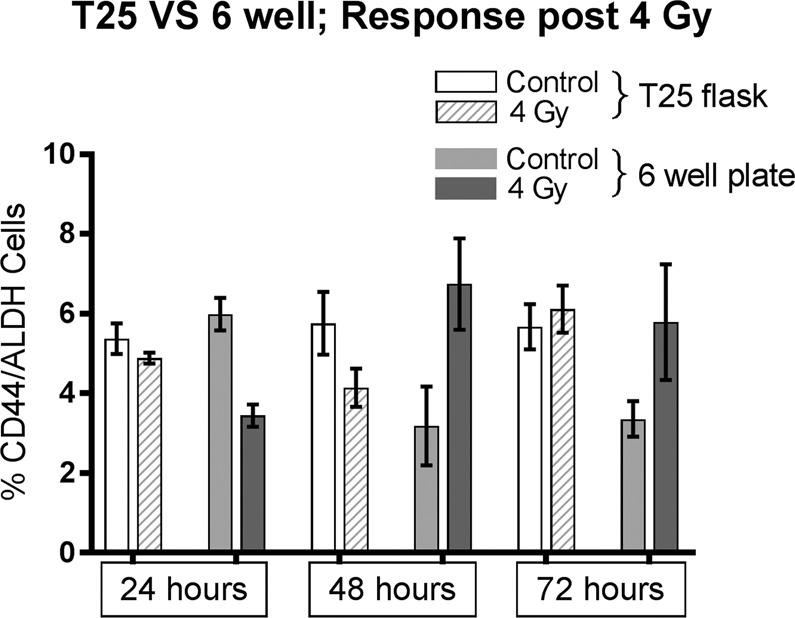
Comparison of CD44+/ALDH + measurements for UM-SCC-47 by T25 and 6 well plate protocols. Analysis repeats n = 7. Error bars by standard error of mean. GraphPad Prism v8.2.1 www.graphpad.com.

To test for contribution of any associative effects upon CD44+/ALDH + measurements resulting for cells remaining *in situ* following irradiation until flow cytometry analysis, a comparison of results from parallel assays was performed to validate the T25 flask protocol (Fig. 5).

The results of concurrent CSC measurements, following a 4 Gy dose, of UM-SCC-47 from T25 and 6 well plate protocols found the only significant difference in results by method was for irradiated cells at 24 hours (Table 2). The results from the 6 well assay showed a greater range of error, likely due to confounding effects of cell handling in this method between treatment and analysis, compared to cells remaining *in situ* using T25 flasks.

**Table 2:** Comparison of means by unpaired t-test for controls or irradiated cells from T25 and 6 well plate methods.

t-test T25 flask vs 6well	24 hours			48 hours			72 hours		
	Sig.	p value	SEM+	Sig.	p value	SEM+	Sig.	p value	SEM+
Control	ns	0.2243	0.5352	ns	0.0644	1.263	ns	0.0666	0.5021
4 Gy irradiation	**	0.0064	0.3425	ns	0.0863	1.239	ns	0.8375	1.567

## Discussion

For locally advanced HNSCC, radiotherapy is a crucial part of the approach to tumour control, but loss of normal tissue function is frequent side effect of this treatment. A relative lack of understanding of the mechanisms and predictability of response to treatment limits attempts to optimise the radiotherapy dose for HPV positive HNSCCs, which exhibit heightened radiosensitivity. CSC proportions of a tumour population, or CSC density, show a correlation with treatment outcomes where higher CSC densities are a negative prognostic biomarker^16,33^. Accordingly, there has been some expectation that the more treatment-responsive HPV positive HNSCCs would display lower CSC densities in keeping with these better clinical outcomes. This expectation was not borne out however by our findings in six HNSCC cell lines, of the baseline proportions of putative CSC populations, which were measured as the the proportion of CD44+/ALDH + cells. Here, the CSC densities among the untreated HNSCC cell lines varied significantly independent of HPV status and these findings are consistent with results found by others. Identifying the CSC phenotype by ALDH alone, Zhang, *et al*.^25^ observed greater CSC density in HPV positive cell lines as well as in tumour samples. This was contrary to findings of lower CSC frequency in HPV positive cell lines by Vlashi, *et al*.^23^, using the marker ZsGreen-cODC positive. Evidence reported by Tang, *et al*.^24^ found no correlation between HPV status and CSC density. Our measures of CSC density by the CD44+/ALDH + phenotype among the untreated HNSCC cell lines, also found no correlation between CSC density and HPV status. These conflicting data suggest that, because these putative CSCs display a similar phenotype, despite having differences in HPV status, HPV may not be an important aetiologic factor in the population density of CSCs. However, what role CSCs might play where significantly different clinical outcomes are observed may not simply be the result of their tumoral density but rather reflect an altered capacity to respond to treatment. Hence, characterising the biological behaviour of tumoral CSCs may better predict response to therapy than measurements of CSC density alone^34^.

To test for differences in the radio-responsiveness of the CD44+/ALDH + phenotype of HPV positive versus negative HNSCC cells, cell cultures were irradiated with a 4 Gy dose and analysed for changes in the proportion of this phenotype over intervals of 24, 48 and 72 hours, reflecting time intervals between fractionated doses in external radiotherapy. Resulting data is novel in that it provides a quantitative comparison of dynamic population responses among HSCC CSCs, by their HPV status, at therapeutically relevant dose and time points following irradiation. Among irradiated cell cultures, only 1 of the 6 cell lines (UM-SCC-22a) showed any increase in CSC density at 24 hours. Although CSC density increased among the HPV positive lines, UPCI-SCC-154 and UPCI-SCC-090, the HPV negative populations of HNSCC cells more often had significantly greater increases in the proportion of CSCs during the 3 days after irradiation. To compare how differences in HPV status might affect the overall extent to which CSC density responded at 24, 38, and 72 hours after irradiation, we calculated the ratio of the proportions of putative CSCs post-treatment to baseline (untreated) cell line populations, at each time point for either the HPV positive or the HPV negative HNSCC cell lines. We found that this ratio increased steadily during the 3 days after irradiation so that the proportional change in CSC density of the irradiated HPV negative HNSCC cells was significantly greater than that of the irradiated HPV positive cells by day 3. The increased proportions of CSCs over the 3 days may indicate that several different biological processes are at work including preferential survival, altered divisional dynamics and/or cellular plasticity by de-differentiation. The danger of clonogen-driven acceleration in tumour repopulation, in response to sublethal or delayed doses of radiation, has been earlier described by Withers, *et al*.^35^. CSCs are shown to typically replicate asymmetrically where the division of a CSC results in a progenitor daughter cell (commencing commitment to differentiation) and another CSC, representing self-renewal. But in response to tumour volume loss from irradiation, cellular signalling may switch the CSC divisional process to a symmetrical one where both daughter cells are CSCs, rapidly increasing their proportion in the tumour population and thus their potential for proliferation^12,36,37^. Furthermore, plasticity among progenitor and differentiated cells, in response to irradiation, can increase CSC populations by dedifferentiation where these cells acquire the CSC phenotype and the capacity for self-renewal^12,13^. Observed increases in CSC proportions demonstrate that CSC numbers are not static but may be responsive to therapeutic levels of irradiation where a significantly greater effect was observed in the HPV negative group. This observed difference suggests a potential difference in radio-responsiveness among HNSCCs depending on aetiology, and warrants further investigation. The fact that recurrent primary HNSCC tumours are the most common cause of mortality points to the importance of our finding that HPV status seems to have differential effects on the relative proportions of putative CSCs among the entire cell population of HNSCCs after a single 4 Gy dose of X-irradiation and thus may also affect the relative repopulating ability of CSCs according to their HPV status^38^.

Comparison of protocols (T25 flask vs 6 well plate) for potential effects on CSC counts, found one significant difference in results from these methods among the 6 comparisons of means. This may be indicative of the extent to which methods can influence this data and needs to be considered in the interpretation of results. The measurement of CSCs from cells remaining *in situ* in T25 flasks demonstrated less range of error than the 6 well plate method and was felt to introduce fewer confounding effect from cell handling.

## Conclusion and Future work

There is very little quantitative data in the scientific literature on the cellular dynamics of cancer stem cells originating from HNSCC, in response to therapeutic levels of X-irradiation, which expressly compares CSCs by HPV status. This work provides additional insight using data from 6 cell lines (3 HPV positive and 3 HPV negative), thus enabling for the first time quantitative comparison between HNSCC aetiologies by analysis of dynamic responses among CSCs to therapeutic levels of radiation in terms of their HPV status. The main highlights of the current work are as follows:

- inherent CSC densities among the investigated HNSCC cell line populations did not associate with HPV status;
- the study compared changes in CSC densities within the cell line population, by HPV status, in response to radiation;
- quantitative differences in CSC populations were shown using 6 cells lines, even amongst HPVpositive and negative sub-groups, based on their origin;
- the behavioural response of putative CSCs among the 6 cell lines investigated demonstrated elevations in CSC densities following a 4 Gy dose that were significantly greater in the HPV negative group, potentially indicating stronger repopulating ability;
- the dynamic quantitative data obtained for the 6 cell lines facillitates progress of knowledge from generic to specific, as only qualitative studies have been reported so far in the literature; this information is important for future *in vitro*, *in vivo* and in silico (modelling studies) that require specific quantitative inputs.

Data on responsiveness of HNSCC clonogens to therapeutic levels of radiation, comparing HPV statuses, is of vital interest in finding better clinical applications. Given the number of clinical trials investigating dose de-escalation for HPV positive HNSCC, quantification of intrinsic capacity for response to treatments by CSCs in both HPV statuses is of critical concern and value to informing approaches toward de-escalation.

The differential response of CSC densities to therapeutic levels of X-ray dose, according to HPV status, warrants further studies. Apoptotic studies and FACS analysis, to measure preferential survival against proliferative of differential changes in phenotype proportions are required to measure their influence in resulting phenotype proportions over time following irradiation. Testing of intrinsic radiosensitivity by proliferative capacity following fractionated irradiation, using clonogenic assays of cell line populations, needs to be part of future work determine dynamic response to repeated dose. Any changes in cell survival, as a function of dose and HPV status, following repeated 4 Gy dose would provide data on changes to intrinsic radiosensitivity reflective of the therapeutic use of radiation.
